# Comparative study of greater occipital nerve block and conventional therapy in chronic migraine

**DOI:** 10.6026/973206300213947

**Published:** 2025-10-31

**Authors:** Kaushal Kabir, Ritu Pauranik, Kirti Agrawal, Manish Banjare

**Affiliations:** 1 Department of Anaesthesia, Mahatma Gandhi Memorial Medical College, Indore, India

**Keywords:** Chronic migraine, greater occipital nerve block, ultrasound-guided, prophylactic therapy, VAS scores, MIDAS score

## Abstract

Chronic migraine is disabling and medicines alone are often not enough so ultrasound guided greater occipital nerve block is used as an add-on therapy. We observed 100 patients where one group received prophylaxis with the block and the other received only prophylaxis with follow up of 90 days. The block group showed a bigger fall in migraine disability score (14.2 to 6.8 vs 14.3 to 9.2 p < 0.0001), lower VAS score (7.9 to 4.4 vs 7.1 to 6.0 p < 0.0001) and fewer headache episodes early and higher satisfaction (12.7 vs 12.3 p = 0.016). Ultrasound guided block was safe and simple and gave better pain relief with reduced medicine use and improved patient satisfaction.

## Background:

Chronic migraine is defined as headache on at least 15 days per month for more than three months with migraine-like symptoms on at least eight of those days [1]. It affects about 2% of general population and nearly 8% of migraine patients leading to major disability in daily life [2, 3]. Management is often tough, drug overuse further worsens condition and economic burden is also high. Preventive choices are few where drugs which work in episodic migraine mostly fail in chronic migraine [4]. Only topiramate and botulinum toxin A have strong evidence but both carry issues of cost, side effects and tolerability [4, 5]. Because of this, interest has shifted to minimally invasive methods like peripheral nerve blocks. Among them greater occipital nerve block is promising [6]. Injection of local anaesthetic with steroid near the nerve reduces inflammation and pain. Using ultrasound guidance makes the block more accurate and successful [7, 8]. Greater occipital nerve block modulates pain pathways involving trigeminocervical complex, which is central in migraine [7, 8, 9, 10- 11]. It is safe, cost-effective and has minimal drug interaction and around two-thirds patients get relief, lasting weeks to months [12]. Therefore, it is of interest to report the outcomes of ultrasound guided greater occipital nerve block in chronic migraine patients already on preventive therapy.

## Materials and Methods:

This prospective clinical study was done between September 2023 - December 2024 after approval from the Institutional Ethics Committee (EC/MGM/SEPT-23/85), Department of Anaesthesiology, Pain Clinic, M.G.M. Medical College and M.Y. Hospital, Indore. The study was single centre, low risk, academic type and not registered in any trial registry. No external funding was taken. Patients with chronic migraine on standard prophylactic therapy referred from Medicine department were screened. One hundred patients aged 18-60 years, ASA grade I or II, and on similar class of prophylactic drugs were included. Exclusion was drug allergy, prior occipital surgery or coagulopathy. Written informed consent was obtained. Patients were randomised by chit method into two equal groups. Group PB received prophylaxis plus ultrasound guided greater occipital nerve block. Group P received prophylaxis only. Baseline vitals recorded and IV line secured. In Group PB, block was given by trained anaesthetist under aseptic precautions using high frequency linear ultrasound probe. A mixture of 2 ml 1% preservative free lignocaine with 4 mg dexamethasone (total 3 ml) was injected at the nerve site. Patients observed for 30 minutes in recovery and one hour in post-op for any side effects. All patients were advised to keep diary of headache attacks and analgesic use. Naproxen sodium 500 mg once daily was prescribed as rescue. Follow up was done at day 7, 30, 60 and 90 in pain clinic, or by phone if not able to visit. Patients lost to follow up were excluded. Severity of headache was measured with Migraine Disability Assessment Score (MIDAS) and Visual Analogue Scale (VAS). Existing prophylaxis drugs were continued without change. Any adverse event was managed as per hospital protocol. Quantitative data with normal distribution was analysed using independent t-test, and non-normal data with Mann-Whitney test. Within group comparisons were done with paired t-test. Qualitative variables were compared with Chi-square. Data was entered in Microsoft Excel and analysed in SPSS v25. A p value less than 0.05 were considered significant.

## Results:

A total of 100 chronic migraine patients were included, 30% male and 70% female. Mean age was almost same in both groups, 31.28 ± 6.01 years in the block group and 30.3 ± 6.35 years in prophylaxis group (p=0.43). Baseline demographic features are shown in Table 1 (see PDF). Baseline MIDAS and VAS were comparable as in Table 2 (see PDF). After treatment, block group showed clear improvement at all follow ups. MIDAS reduced to 6.48 ± 0.81 at day 7, 6.54 ± 0.61 at day 30, 6.74 ± 0.75 at day 60 and 6.8 ± 0.78 at day 90 while prophylaxis group stayed higher with scores 9.54 ± 1.2, 9.06 ± 1.53, 9.2 ± 1.36 and 9.22 ± 1.17 (all p<0.0001). Detailed comparison is given in Table 2 (see PDF). VAS also dropped more in block group reaching 4.6 ± 0.53 at day 7 and 4.48 ± 0.5 at day 90, compared to 6.38 ± 0.67 and 6.02 ± 0.68 in prophylaxis group (p<0.0001). These results are shown in Table 3 (see PDF). Patient satisfaction was better with the block, mean score 12.72 ± 0.78 compared to 12.32 ± 0.84 (p=0.016). Headache frequency too was less in block group during early follow up where the mean 2.82 ± 0.75 at day 7, 5.2 ± 0.7 at day 30 and 7.6 ± 0.67 at day 60 the prophylaxis group had 3.8 ± 0.99, 5.66 ± 1.04 and 8.18 ± 0.75 respectively. By day 90 difference narrowed here block group had 8.32 ± 0.65 and prophylaxis group was 8.48 ± 0.61 (p=0.21). No major adverse event was reported.

## Discussion:

Chronic migraine is disabling, it affects daily life, work productivity and increases treatment cost. Drug options and non-drug methods both have been tried, but greater occipital nerve block is gaining more attention because it is simple, minimally invasive and gives early relief. The main mechanism is through trigeminocervical complex, where greater occipital nerve plays central role in pain transmission [13, 14]. This nerve connects to trigeminal nucleus caudalis, which is major site for processing headache signals [15]. Injecting local anaesthetic with steroid around this nerve interrupts the pathway, reduces frequency and severity of attacks [16]. Ultrasound guidance adds accuracy, reduces operator variability and improves safety. Several studies support the role of this block in chronic migraine. Afridi *et al.* used lidocaine and methylprednisolone in 54 patients, and reported 15.8% complete response and 29.8% partial relief at 4 weeks, with about 9 days of total pain freedom and median 30 days partial relief [17]. Weibel *et al.* studied 150 patients with cervicogenic features, used bupivacaine and triamcinolone and showed that 52% had more than 50% reduction in headache days after one month [18]. Gürsoy *et al.* compared ultrasound guided and landmark technique, and found ultrasound guidance gave better results with lower VAS and fewer attacks [19]. Our findings are in line with these again showing the value of ultrasound precision. Proper technique is essential where Greher *et al.* described two sonographic methods: one at superior nuchal line near occipital artery and another at C2 level over obliquus capitis inferior muscle [20]. We followed the C2 level approach, separating hair, applying sterile gel and using linear probe for clear image and exact needle placement. This technique increases reliability and reduces block failure. In our study the patients who received nerve block plus prophylaxis had better outcomes compared to prophylaxis only. MIDAS scores dropped significantly at all follow ups (day 7, 30, 60, 90; p < 0.0001) showing disability improvements. VAS scores were also consistently lowers a confirming superior pain control. Patient satisfaction was higher (p = 0.016). Use of rescue analgesic was less in block group, which matches earlier reports by Cuadrado *et al.* and Ulusoy & Bolatturk [21, 22]. Recent reviews and trials further strengthen the evidence. Arata *et al.* in a narrative review of occipital nerve blocks for headache found consistent short-term benefit across migraine, cluster and occipital neuralgia with most patients reporting 2-6 weeks relief and only minor adverse events [23]. Gürsoy and Tuna [19] in their 2024 study of 60 chronic migraine patients showed ultrasound guided block decreased mean monthly headache days from 15.2 to 8.1 at three months, while landmark block reduced from 14.7 to 10.9, confirming superiority of ultrasound technique. Katalinic *et al.* also emphasised that the block is safe, low cost and effective with a relief often lasting several weeks after single injection [24]. They suggested limiting to maximum three injections in six months, after which other strategies should be considered. The drug combination of 2 ml preservative free lignocaine with 4 mg dexamethasone was effective and safe with no major adverse event reported. This study has small sample size, so results cannot be generalised. Larger multicentre studies are needed for stronger evidence. Follow up was only for ninety days, so long-term effect and ideal interval for repeat block remains uncertain. Even though greater occipital nerve block looks cost-effective compared to botulinum toxin A, proper economic evaluation is still required before recommending its routine use.

## Conclusion:

Ultrasound guided greater occipital nerve block is simple, safe and effective for chronic migraine. It gives better pain relief, lowers disability and reduces need of rescue drugs when added to prophylaxis. Short term benefit is clear, but long term role and cost effectiveness need more study.
